# Decoding resistance: a comprehensive study on pathogens and antibiotic efficacy in periprosthetic joint infections

**DOI:** 10.1186/s13018-026-07165-9

**Published:** 2026-08-22

**Authors:** Niklas Hahn, Marcus Jäger, Alexander Wegner, Tobias Ludger Schulte, André Busch

**Affiliations:** 1 Department of Infectious Diseases and Nephrology, West German Centre of Infectious Diseases, University Hospital Essen, 45147 Essen, Germany; 2 Department of Orthopedics, Trauma and Reconstructive Surgery, Katholisches Klinikum Essen Philippus, 45355 Essen, Germany; 3 Department of Orthopedics, Trauma and Reconstructive Surgery, St. Marien Hospital Mülheim a. d. Ruhr, 45468 Mülheim, Germany; 4 Chair of Orthopedics and Trauma Surgery, University of Duisburg-Essen, 45147 Essen, Germany; 5 Department of Orthopedics and Trauma Surgery, Katholisches Klinikum Bochum - St. Josef Hospital, Ruhr University Bochum, Bochum, Germany

**Keywords:** Periprosthetic joint infection, Antibiotic therapy, Resistance

## Abstract

**Background:**

Periprosthetic joint infection (PJI) continues to be a severe complication following total joint arthroplasty (TJA). Identifying the causative pathogens and their antibiotic resistance profiles is essential for the effective eradication.

**Materials and methods:**

In this retrospective study, 825 antibiograms and demographic data of patients with confirmed PJIs from 2005 to 2021 were evaluated. The resistance profile evaluation focused on the antibiotics ampicillin/sulbactam, clindamycin, gentamicin, vancomycin, ciprofloxacin and rifampicin.

**Results:**

Methicillin-sensitive *staphylococcus aureus* (MSSA) was the most prevalent pathogen (20.8%) followed by methicillin-resistant *staphylococcus epidermidis* (MRSE, 16.1%) and methicillin-sensitive *staphylococcus epidermidis* (MSSE, 10.8%). Polymicrobial (PM) PJI (15.9%) was also high with MSSA (21.2%) as the most frequent pathogen followed by gram-negative (GN) pathogens (20.2%) and MRSE (18.3%). The overall resistance rate against ampicillin/sulbactam and clindamycin was 42.3% and 40%, respectively. Vancomycin showed low resistance rates of nearly 1% with higher rates in *enterococci* (4%). MSSA and MRSA exhibit high sensitivity rates against gentamicin (97.4% and 89.5%). Gentamicin resistance was high in *staphylococcus epidermidis* (43%) and particularly in MRSE (64.6%). Ciprofloxacin resistance was especially high among MRSA (81.3%) and MRSE (84.0%) isolates, whereas sensitivity was most common in GN (78.5%) and MSSE (85.0%) groups.

**Summary:**

This study reveals high rates of MRSE, SE and GN pathogens causing PJI. GN and MRSE rates were also increased in PM PJI. The high overall resistance rates against ampicillin/sulbactam raise doubts as first line empiric oral antibiotic in PJI. The nearly 20% ciprofloxacin resistant GN pathogens should be alarming due to limited choice of biofilm-penetrating antibiotics in GN PJI.

## Introduction

Rising life expectancy in the context of demographic change leads to an increasing number of arthroplasty interventions in orthopaedics [1–3] Periprosthetic joint infections (PJI) are serious complications after TJA associated with morbidity.

[4] Although preventive measures and adequate antibiotic therapies have reduced the rates of PJI [5], a proportional increase in PJIs is expected as the number of prosthesis implantations increases [6]. PJI can be caused by a variety of pathogens appearing in both monobacterial and polymicrobial infections [7]. *Staphylococcus epidermidis* and *Staphylococcus aureus* are the most common causative pathogens with rates between 50 and 60% [8].

Besides surgical intervention antibiotic treatment is an essential part of therapy for PJI intending to eradicate the causative pathogens. Due to thermal instability only gentamicin, vancomycin and clindamycin are usually used in antibiotic loaded bone cement spacers (ALBCS) [9]. However, ALBCS used in two stage revision often become colonized by bacteria due to rapid decrease of local antibiotic concentration, allowing new biofilm formation [10]. In addition, not all antibiotics can be manually admixed in bone cement spacers because they need to be hydrophilic and thermostable [10]. Therefore, the systemically applied antibiotic treatment is a key for successful antibiotic treatment in PJI. To optimize the systemic antibiotic treatment of PJI the surgeon must have knowledge of the causative pathogen as well as the antibiotic susceptibility. If the pathogen is unknown, the surgeon must rely on recommendations [11]. Ampicillin/sulbactam in particular is used for systemic empirical therapy, as previously described to be effective against a broad spectrum of bacteria and can also be used for septic patients, multiple previous operations and suspected low-grade infections [10].

The growing threat of antibiotic-resistant strains makes the management of PJIs increasingly complex, a development that weakens the effectiveness of conventional antibiotics. For example, methicillin-resistant *staphylococcus aureus* (MRSA) PJI shows a higher risk of treatment failure and poorer prognosis [12].

The present study aims to reveal pathogen spectrum and resistance situation in two german endoprosthesis centers.

## Materials and methods

After approval by the review board of the University of Duisburg-Essen (19-8609-BO), a retrospective study was performed of data gathered from the Departments of Orthopedics and Trauma Surgery from University of Duisburg-Essen and Bochum Germany. All data with ICD coding T84.5 between 2005 and 2021 were included in the study. We analyzed 825 antibiograms of 663 patients suffering from PJI. PJI was diagnosed according to MSIS criteria if pathogens were detected in two intraoperatively gathered specimens or histology demonstrated infection. Analysis focused on pathogen identification, surgical site, patient age, gender, and resistance status.

Antibiograms were generated using microbiological tests to determine infectious pathogens and the minimum inhibitory concentration (MIC), representing the concentration of an active substance necessary to prevent pathogen growth in a culture. Results were stratified according to EUCAST (European Committee on Antimicrobial Susceptibility Testing) breakpoints into categories: sensitive (S), intermediate (I), and resistant (R).

Pathogen identification and resistogram preparation were conducted utilizing MALDI-TOF MS (matrix-assisted laser desorption/ionization time-of-flight mass spectrometry) technology from bioMérieux and Bruker. Bacterial metabolic performance was evaluated through the microdilution method using “Vitek 2 XL” from bioMérieux and “MicroScan” from Beckman Coulter. These same microdilution methods were employed to construct the resistograms. For isolating low-virulent anaerobic organisms (e.g. Cutibacterium acnes) special protocols were applied. Strict anaerobiosis using an anaerobic chamber (gas phase: 80% N_2_, 10% CO_2_, 10% H_2_) with incubation temperature of 37° C was applied for 14 days was applied.

Data were organized based on individual pathogen identification and patient cases, facilitating analysis of specific pathogen profiles and patient-associated infection patterns. The number of cases varied depending on the sorting method; for instance, polymicrobial PJIs were consolidated into single cases when sorting by patient cases.

### Statistics

Descriptive statistics, alongside absolute and relative frequencies for categorical variables, were employed to characterize key data attributes. Chi^2^ tests, t-tests, and ANOVA were utilized to explore inter-variable relationships, contingent on their scale level and distributional assumption. The analysis presumed normal distribution, absence of outliers, and homogeneity of variance, with correlation analyses employed to demonstrate relationships between metric variables. Statistical tests such as the Shapiro–Wilk test for normal distribution and the Levene test for variance homogeneity were conducted, complemented by graphical techniques. Non-parametric tests were applied for violations of assumptions (Games-Howell test, Kruskal–Wallis test). Effect sizes were utilized to evaluate the significance of findings, while odds ratios were computed to ascertain event probability ratios. Odds ratios, which represent the relationship between the occurrence of an event (infection) and its absence, were used to assess the risk of infection depending on the surgical site and pathogen type. All analyses were conducted using SPSS with a significance level of 5%.

## Results

A total of 825 resistograms in 663 patients were evaluated. There were 56.6% (n = 375) were female, and 43.4% male (n = 288) patients. The median age was 72.1 years (30–97). Regarding the detected surgical areas 659 valid observations were recorded. Predominantly, PJIs involved the hip (60.4%, n = 398), followed by the knee (34.6%, n = 228), and the shoulder (5%, n = 33).

A total of 627 pathogen observations were documented, with methicillin-sensitive *Staphylococcus aureus* (MSSA, 20.8%) being the most prevalent. *Staphylococcus epidermidis*, either methicillin-resistant (MRSE, 16.1%) or methicillin-sensitive (MSSE, 10.8%) were the causative pathogens for PJI. *Streptococci* or *enterococci* (SE) were found in 76 cases (11.5%). Gram- negative (GN) pathogens were detected in 13% (n=86). In 105 cases (15.9%) a polymicrobial (PM) infection was present. No pathogens (Culture-negative, CN) were detected in 33 (5.0%) cases (Table 1).

**Table 1:** Pathogen distribution of PJI cases

Pathogen	Absolute frequency	Relative frequency (%)
Methicillin-sensitive *Staphylococcus aureus* (MSSA)	137	20.8
Methicillin-resistant *Staphylococcus aureus* (MRSA)	20	2.8
Methicillin-sensitive *Staphylococcus epidermidis* (MSSE)	71	10.8
Methicillin-resistant *Staphylococcus epidermidis* (MRSE)	106	16.1
*Streptococci* and *Enterococci* (SE)	76	11.5
Gram-negative (GN)	86	13.0
Other	26	3.9
Polymicrobial (PM)	105	15.9
Culture-negative (CN)	33	5.0

In the presence of PM PJI, MSSA (21.2%) was the most frequent pathogen followed by GN (20.2%) and MRSE (18.3%). SE, MSSE and MRSA were found in 14.7, 11.5 and 2.8%, respectively. Other pathogens were involved in PM PJI in 11.2%.

MSSA was most frequently detected in knee infections (24.2%), followed by the hip (18.9%) and shoulder (15.2%). MSSE and SE were more commonly identified in knee joint infections compared to other locations. GN pathogens were predominantly found in the hip (16.2%) and less frequently in the knee (8.4%) and shoulder (6.1%). Polymicrobial infections showed a similar distribution pattern, with the highest frequency at the hip (16.9%) and lower proportions at the knee (15.0%) and shoulder (12.1%).

MRSA was evenly distributed across sites, but with slightly higher occurrence at the shoulder (2.9%) compared to hip (2.5%) and knee (2.6%). MRSE infections were recorded across all locations, with a relatively high proportion at the shoulder (18.2%) compared to the hip (16.4%) and knee (15.4%). Notably, infections caused by other gram-positive rods (GPR) were most prevalent in the shoulder region (15.2%), compared to lower detection rates at the hip (4.5%) and knee (1.3%). CN findings were recorded in all regions, but were proportionally more common in shoulder infections (9.1%) than in hip (3.5%) and knee (7.0%) cases (Table 2).

**Table 2:** Pathogen distribution by surgical site

Pathogen	Hip	Knee	Shoulder
MSSA	75 (18.9%)	55 (24.2%)	5 (15.2%)
MRSA	10 (2.5%)	6 (2.6%)	3 (2.9%)
MSSE	38 (9.6%)	29 (12.8%)	4 (12.1%)
MRSE	65 (16.4%)	35 (15.4%)	6 (18.2%)
SE	45 (11.4%)	30 (13.2%)	1 (3.0%)
GN	64 (16.2%)	19 (8.4%)	2 (6.1%)
GPR	18 (4.5%)	3 (1.3%)	5 (15.2%)
PM	67 (16.9%)	34 (15.0%)	4 (12.1%)
CN	14 (3.5%)	16 (7.0%)	3 (9.1%)

### Pathogen distribution over time

**Fig. 1:**
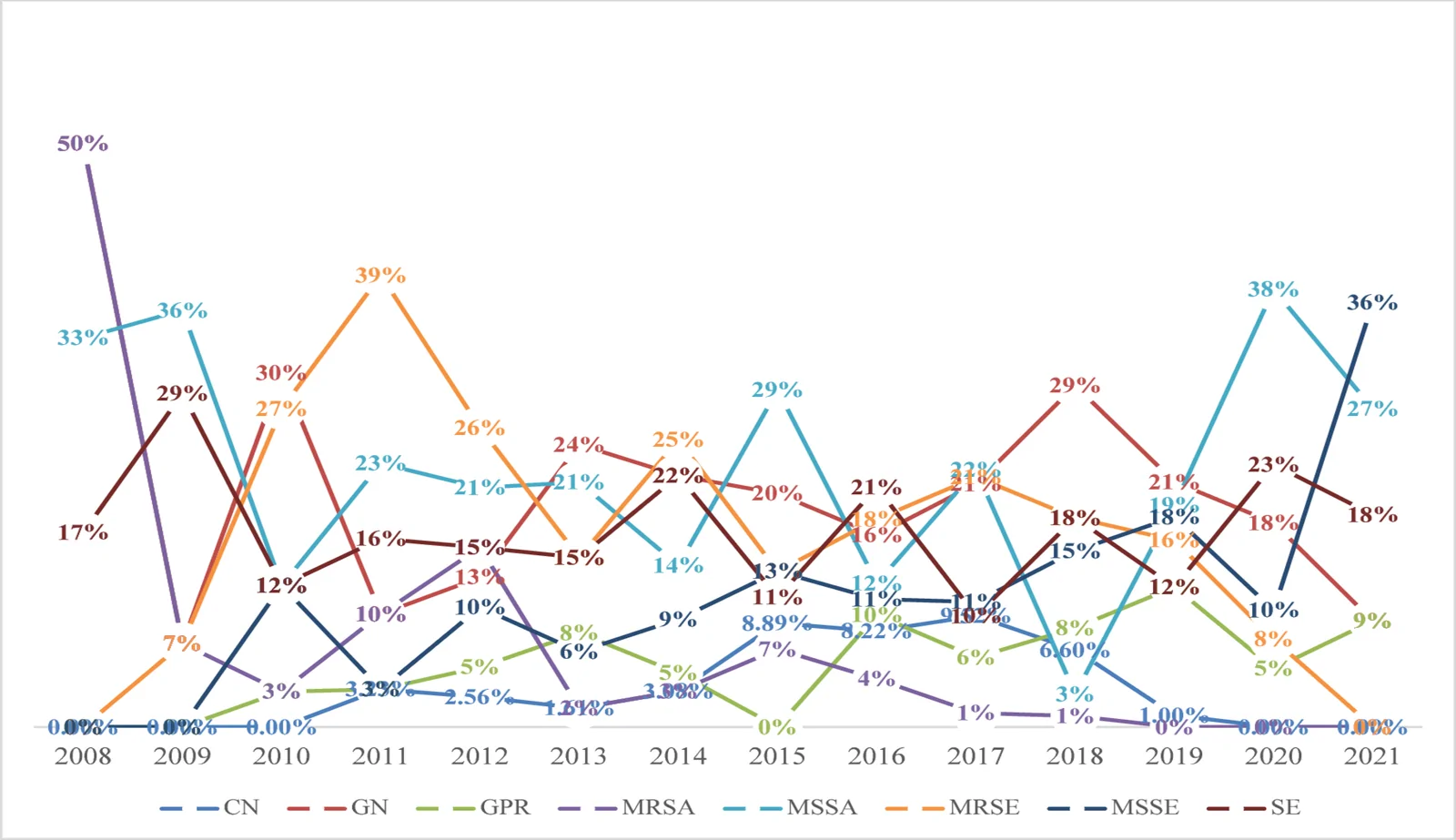
Pathogen distribution over time

The percentage distribution of the pathogens for each year is illustrated in Fig. 1. The largest increased was oberserved among MSSE (r^2 ^= 0.451), GN (r^2 ^= 0.317) and SE (r^2 ^= 0.441). Moderate increase was detected in GPR (r^2 ^= 0.371), MSSA (r^2 ^= 0.294) and MRSE (r^2 ^= 0.208). Almost no increase was found in MRSA (r^2 ^= 0.015).

### Risk for infection with specific pathogen correlates with surgical site

In hip joints, gram-negative infections are 2.2 times, polymicrobial infections 1.2 times and infections with other gram-positive rods (GPR) 1.5 times more frequent than at other surgical sites. The likelihood of MSSA and MSSE infection is 1.4 times higher at the knee compared to other surgical sites.

**Fig. 2:**
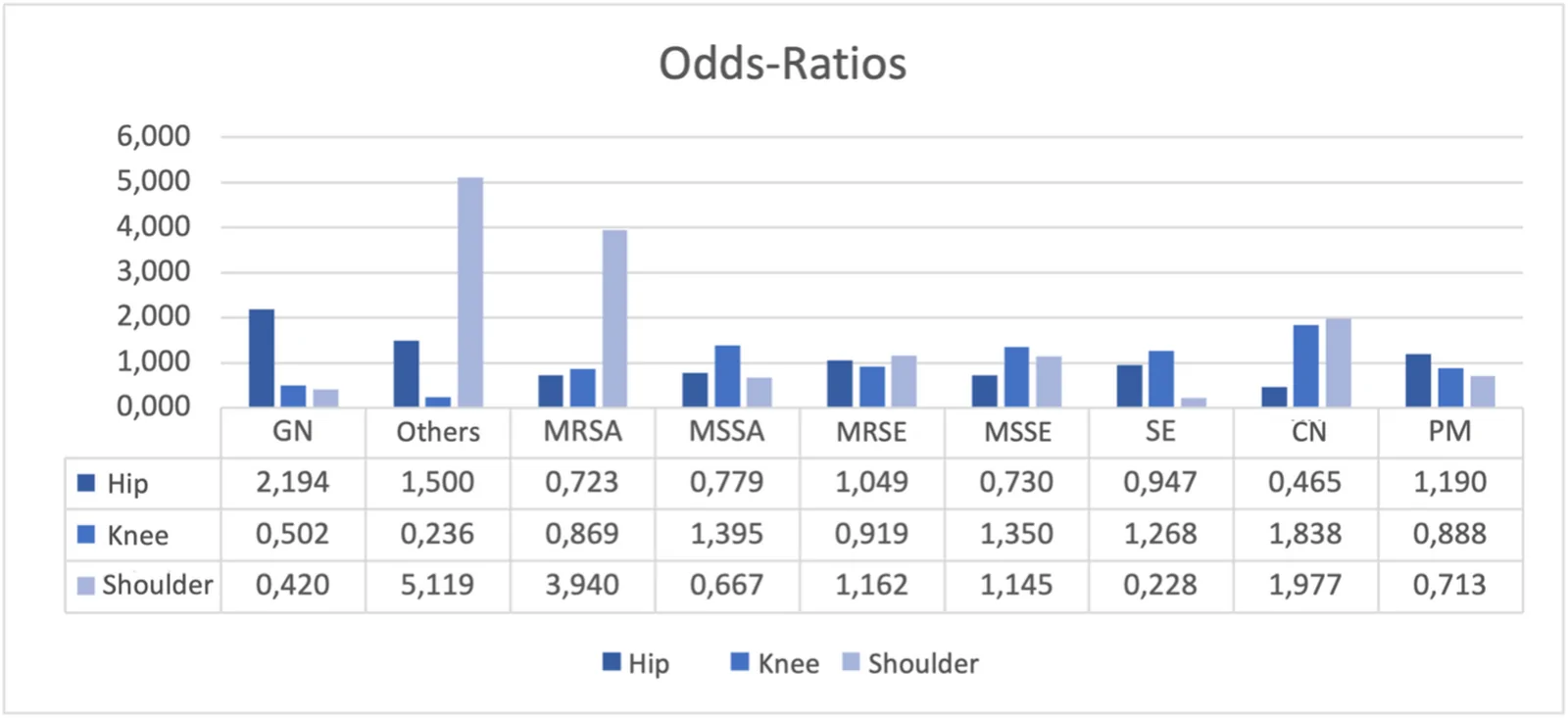
Odds-ratios for pathogen infection by surgical site

Regarding other gram-positive rods (Other) infections, the risk is 5.2 times higher for shoulder infections, notably with *cutibacterium acnes* exhibiting a sub-analysis risk of 9.6 in comparison to other areas. MRSA infections are 3.9 times more frequent at the shoulder, while infections with MRSE and MSSE pose a 1.2- and 1.1 times elevated risk, respectively, compared to other localisations (Fig. 2).

### Overall resistance over time

On average, the statistical resistance rate decreased by about 2.38 percentage points per year during the recorded period (*p* = 0.000076). The coefficient of determination for the linear trend of the resistance rate over the entire period from 2008 to 2021 is r^2^ = 0.7418.

### Resistance of PJI-pathogens regarding first line antibiotic therapy

A total of 480 cases were examined regarding resistance to ampicillin/sulbactam. Sensitivity was observed in 54.6% of cases, while 42.3% exhibited resistance, and intermediate resistance was detected in 3.1% of cases. The Chi^2^ test confirmed a significant correlation between pathogen groups and resistance (χ^2^(12) = 327.02, *p* < 0.01). Notably, MRSA (91.7%) and MRSE (100%) exhibited particularly high resistance rates. Among GN microorganisms (n = 118), the majority comprised *enterobacteria* (n = 104) and *pseudomonads* (n = 11). Ampicillin resistance was observed in 56.7% of *enterobacteria* and 90.9% of *pseudomonads* (Table 3). A weak linear reduction of resistance was found for ampicillin/sulbactam (r^2 ^= 0.237) over observation time.

**Table 3:** Resistogram Ampicillin/Sulbactam

Pathogen	Ampicillin/Sulbactam		
	R	S	I
GN	71 (60.2%)	33 (28.0%)	14 (11.9%)
Others	5 (35.7%)	9 (64.3%)	0 (0.0%)
MRSA	11 (91.7%)	1 (8.3%)	0 (0.0%)
MSSA	6 (5.4%)	105 (94.6%)	0 (0.0%)
MRSE	95 (100.0%)	0 (0.0%)	0 (0.0%)
MSSE	2 (4.0%)	48 (96.0%)	0 (0.0%)
SE	13 (16.3%)	66 (82.5%)	1 (1.3%)
Total	203 (42.3%)	262 (54.6%)	15 (3.0%)

In the assessment of 513 cases for clindamycin, sensitivity was observed in 59.6% of pathogens, while resistance was noted in 40.4%. Significant differences between pathogen groups were confirmed by the Chi^2^ test (χ^2^(12) = 149.72, *p* < 0.01). MRSA (76.2%) and MRSE (75.2%) exhibited notably high resistance rates. Resistance rates were comparatively lower for GN and “Others” pathogens (28.6 and 32.4%, respectively). Within the SE group, *enterococci* dominantly contributed to the high resistance rate (100%) (Table 4). A moderate linear downward trend (r^2 ^= 0.3868) was shown for clindamycin over time.

**Table 4:** Resistogram clindamycin

Pathogen	Clindamycin		
	R	S	I
GN	2 (28.6%)	5 (71.4%)	0 (0.0%)
Other	12 (32.4%)	25 (67.6%)	0 (0.0%)
MRSA	16 (76.2%)	5 (23.8%)	0 (0.0%)
MSSA	19 (11.9%)	139 (87.4%)	1 (0.6%)
MRSE	100 (75.2%)	33 (24.8%)	0 (0.0%)
MSSE	20 (23.0%)	66 (75.9%)	1 (1.3%)
SE	36 (52.2%)	33 (47.8%)	0 (0.0%)
Total	205 (40.0%)	306 (59.6%)	2 (0.4%)

A total of 562 gentamicin resistance tests were conducted, with sensitivity observed in 70.6% of pathogens and resistance in 28.6%. Significant differences in resistance were confirmed by the Chi^2^ test (χ^2^(12) = 261.99, *p* < 0.001). High sensitivity rates were observed for MRSA (89.5%) and MRSE (64.6%). Resistance was noted in 43% of all *staphylococcus epidermidis*. Notably, GN pathogens exhibited a high sensitivity of 82.3%, while *streptococci* and *enterococci* displayed elevated resistance rates (90.7%) (Table 5). A weak linear downward trend was detected for gentamicin resistance over time (r^2 ^= 0.2115).

**Table 5:** Resistogram gentamicin

Pathogen	Gentamicin		
	R	S	I
GN	18 (14.5%)	102 (82.3%)	4 (3.2%)
Other	6 (60.0%)	4 (40.0%)	0 (0.0%)
MRSA	2 (10.5%)	17 (89.5%)	0 (0.0%)
MSSA	4 (2.6%)	148 (97.4%)	0 (0.0%)
MRSE	84 (64.6%)	46 (35.4%)	0 (0.0%)
MSSE	8 (9.5%)	76 (90.5%)	0 (0.0%)
SE	39 (90.7%)	4 (9.3%)	0 (0.0%)
Total	161 (28.6%)	397 (70.6%)	4 (0.7%)

A total of 534 cases were examined for vancomycin resistance, with 98.9% of pathogens showing sensitivity and 1.1% exhibiting resistance. Significant correlations between pathogen groups and resistance status were confirmed (χ^2^(5) = 12.083, *p* < 0.05). Most Gram-positive pathogens were sensitive, while among SE pathogens, 3.8% were resistant, primarily identified as vancomycin-resistant *enterococci* (VRE). Notably, GN pathogens were not tested for vancomycin resistance due to inherent resistance (Table 6). Almost no linear trend was seen for vancomycin resistance over time. (r^2 ^= 0.0692).

**Table 6:** Resistogram vancomycin

Pathogen	Vancomycin	
	R	S
Other	0 (0.0%)	29 (100.0%)
MRSA	0 (0.0%)	20 (100.0%)
MSSA	0 (0.0%)	158 (100.0%)
MRSE	1 (0.7%)	134 (99.3%)
MSSE	0 (0.0%)	84 (100.0%)
SE	4 (3.8%)	101 (96.2%)
Total	5 (0.9%)	526 (99.1%)

A total of 457 cases were examined for ciprofloxacin resistance, with 61.3% of pathogens showing sensitivity and 37.4% exhibiting resistance. Significant correlations between pathogen groups and resistance status were confirmed (χ2(12) = 167.79, *p* < 0.001). Resistance was particularly high among MRSA (81.3%) and MRSE (84.0%) isolates, whereas sensitivity was most common in GN (78.5%) and MSSE (85.0%) groups (Table 7). A moderate decline was found in ciprofloxacin resistance over time (r^2 ^= 0.4765).

**Table 7:** Resistogram ciprofloxacin

Pathogen	Ciprofloxacin		
	R	S	I
GN	26 (19.3%)	106 (78.5%)	3 (2.2%)
Other	3 (37.5%)	5 (62.5%)	0 (0.0%)
MRSA	13 (81.3%)	3 (18.7%)	0 (0.0%)
MSSA	12 (11.8%)	87 (85.3%)	3 (2.9%)
MRSE	84 (84.0%)	16 (16.0%)	0 (0.0%)
MSSE	6 (15.0%)	34 (85.0%)	0 (0.0%)
SE	27 (48.2%)	29 (51.8%)	0 (0.0%)
Total	171 (37.4%)	280 (61.3%)	6 (1.3%)

A total of 367 cases were examined for rifampicin resistance, with 93.7% of pathogens showing sensitivity and 5.7% exhibiting resistance. Significant associations between pathogen groups and resistance status were observed (χ2(12) = 35.68, *p* < 0.001). Resistance was mainly detected among MRSE isolates (13.6%), while all GN, MSSE, and other pathogens were fully sensitive. Intermediate resistance was rare and observed only in single cases among MRSE and SE (Table 8). No linear trend was seen for rifampicin resistance over time (r^2 ^= 0.0229).

**Table 8:** Resistogram rifampicin

Pathogen	Rifampicin		
	R	S	I
GN	0 (0.0%)	5 (100.0%)	0 (0.0%)
Other	0 (0.0%)	11 (100.0%)	0 (0.0%)
MRSA	2 (11,8%)	15 (88.2%)	0 (0.0%)
MSSA	3 (2.3%)	126 (97.7%)	0 (0.0%)
MRSE	15 (13.6%)	94 (85.4%)	1 (1.0%)
MSSE	0 (0.0%)	81 (100.0%)	0 (0.0%)
SE	1 (7.1%)	12 (85.7%)	1 (7.1%)
Total	21 (5.7%)	344 (93.7%)	2 (1.0%)

## Discussion

The targeted therapy for PJIs remains a subject of debate and ongoing investigation. The increase in antimicrobial resistance represents a growing challenge for healthcare system, exacerbated by the overuse of antibiotics in both human and animal populations in recent years [13]. Factors such as the aging population, multiple comorbidities, and the emergence of resistant microorganisms elevate the risk of PJI following implantation [1, 14].

In this context, understanding the epidemiology, prevalence, and antibiotic resistance profiles of causative pathogens is essential for guiding empirical treatment strategies for acute PJIs and preventing reinfection, thus potentially reducing the economic burden of revision surgery [3].

Consistent with prior research, our study reaffirms that *staphylococcus epidermidis* (30.2%) and *staphylococcus aureus* (24.1%) predominate among PJI-causing pathogens [15–17]. The incidence of periprosthetic joint infection (PJI) of due to methicillin-resistant bacteria was described to be increasing in the last decade [9]. However, in recent studies the prevalence of MRSA was described to remain at low levels [11, 18]. MRSA-infected prostheses continue to pose significant challenges due to heightened morbidity, mortality, and increased healthcare costs [19, 20]. In our study, fortunately, the MRSA PJI rates with 2.8% remains on a low level. Notably, MRSA PJI were 3.9 times more probable at the shoulder compared to other surgical sites.

Another emerging problem is MRSE PJI. The data about prevalence of MRSE PJI is rare. Bjerke-Kroll described a rate of nearly 9% of MRSE PJI [21]. MRSE often show high rates of resistance to levofloxacin, tetracycline, gentamicin, cotrimoxazole, and clindamycin which are commonly used in the treatment of PJI [22]. Hischebeth et al. (2019) could show a significantly lower rate of patients graded as “definitively free of infection” in 2-stage revision for MRSE PJI compared to patients with infections caused by MSSA, MSSE, and MRSA [23]. In line with previous investigations (Fröschen et al., 2022), our study reveals that *staphylococcus epidermidis* (MSSE + MRSE) and *staphylococcus aureus* are most detected in hip PJIs (26 and 20.4%, respectively). However, the rate of MRSE PJI with around 17% should be alarming. Statistical analysis did not reveal any predominant surgical site of MRSE PJI.

*Streptococci* and *enterococci* collectively accounted for 11.5% of pathogens, aligning with findings from Bjerke-Kroll et al. [21]. *Enterococci* are especially problematic with higher rates of treatment failure due to their ability to form biofilms and their robust resistance mechanisms [24, 25]. Twenty-six to 64% of PJI with *enterococci* are polymicrobial infections [26, 27]. The debate about the treatment strategies in *enterococccal* PJI is controversial. Treatment recommendations vary widely greatly and originate from in vitro and experimental studies or are derive from the guidelines for treatment of infective endocarditis. In penicillin-susceptible isolates aminopenicillin derivates, whereas in penicillin-resistant *enterococci* vancomycin and linezolid are recommended [28]. In recent years, multidrug-resistant organisms, especially vancomycin-resistant *enterococcus* (VRE) and carbapenem-resistant *enterobacteriaceae* (CRE) strains have become more prevalent worldwide, and represent a serious public health threat since they are becoming increasingly resistant to current treatment modalities [29]. *Streptococcal* prosthetic joint infections are also associated with high remission rates [30]. On the other hand, *streptococcus agalactiae* (i.e., hemolytic group B *streptococci*) is generally considered to be associated with a poorer outcome than the other types of streptococcal PJIs [31]. In our study, the rate of *streptococcal* and *enterococcal* PJI´s is with 15% nearly according to the previous literature [32].

Cutibacterium acnes (CA) is a gram-positive rod and a known skin commensal. This organism resides in the pilosebaceous gland and is commonly implicated in the skin condition of acne vulgaris. CA has gained recent attention due to its presence in prosthetic joints, particularly in shoulder surgeries and replacements [33]. Several studies investigating the risk factors for an PJI with CA could show association with male sex [34], presence of hair [35], previous shoulder steroid infection [36] and previous shoulder surgery [37]. Patients suffering from PJI caused by CA often show subclinical symptoms leading to delayed diagnosis [38]. CA is increasingly recognized as a cause of PJI following THA. Şahin R et al. (2025) could demonstrate that nearly half of all Cutibacterium PJIs were associated with the DAA [39].

In recent years, gram-negative (GN) pathogens also gain attention. GN pathogens show heterogeneous resistance mechanisms (extended-spectrum beta-lactamases (ESBL)) posing serious challenges in PJI treatment [40]. In a study by Bjerke-Kroll et al. (2014) with around 7% PJI caused by GN pathogens the rates are considered to be relatively low. However, Fröschen et al. (2022) revealed with around 21% three times higher rates of GN PJI [11] similar to 20% accounted in fracture related infections [41]. Patients revised for GN PJIs are at increased risk of reinfection as opposed to GP infections [42]. Our study also demonstrates a notable prevalence of GN pathogens, accounting for 13% of all pathogen detections. Infections with GN pathogens were 2.2 times more probable to occur at the hip compared to other surgical sites.

Limited data are available regarding the risk factors and outcome of polymicrobial prosthetic joint infection (PJIs) when compared with monomicrobial PJI. Risk factors for polymicrobial PJI include host factors such as obesity, diabetes, and peripheral vascular disease [43]. Polymicrobial hip PJI has been traditionally considered a risk factor for treatment failure in hip revision arthroplasty [44]. The previous literature has reported a polymicrobial incidence of 4–27% of all PJI [45]. Marculescu et al. (2008) described a rate of 19% polymicrobial infections with reduced success of treating at 2 years in polymicrobial PJI (polymicrobial 63.8% vs monomicrobial 72.8%) [46]. In our study, polymicrobial infections constitute a proportion 15.9%.

Despite ongoing research in PJI, a substantial number of patients who are ultimately diagnosed with PJI have negative cultures pre- or intraoperatively, resulting in the clinical entity “culture-negative (CN)-PJI [47]. The prevalence of CN PJI has been reported to range between 0 and 42.1% [48]. Identification of microorganisms causing PJI is an important task for selection of appropriate treatment options and prognosis prediction. Therefore, CN PJI is considered as an important clinical issue. Some studies have demonstrated that there are no significant differences in clinical characteristics between culture-positive (CP) PJI and CN PJI. Diagnosis of CN PJI is difficult and often delayed [49]. In our study, the rate of CN-PJI´s with 5% can be considered as low. This is maybe due to improved laboratory techniques. Empirical treatment with vancomycin combined with cephalosporins or fluoroquinolones has been reported to achieve good results in patients with culture‐negative PJI [50].

Empirical antibiotic therapy in the early phase often relies on guidelines considering regional and national resistance patterns. In Germany, for instance, the “Trampuz therapy scheme” recommends ampicillin/sulbactam as empiric antibiotic therapy for suspected PJIs due to its broad efficacy against gram-negative and gram-positive pathogens [10]. However, recent studies including our study revealed increasing rates of oxacillin-resistant coagulase-negative *staphylococci* [11]. Despite the linear reduction over time, the overall resistance rate against ampicillin/sulbactam of 42.3% in our study has to be considered as very high.

Clindamycin stands out as an effective option against gram-positive pathogens, including *staphylococci*. Clindamycin is a standard preoperative prophylactic antibiotic as second-line agent in case of allergy to cephalpsporines [51]. Clindamycin was shown to be effective in treatment of osteomyelitis, has excellent bioavailability and penetrates in high levels into synovial fluid and bone [52]. However, clindamycin acts only bacteriostatic in the standard dosage [53]. Clindamycin is used as systemic as well as content of ALBCS. In PJI In a recent published study, the PJI rate was shown to be significantly higher using clindamycin as prophylactic antibiotic agent compared to cefazolin (7.2 vs. 3.5%, *p* = 0.042) [54]. Nevertheless, clindamycin is frequently used to treat PJI as a component in ALBCS. Through a high initial peak concentration of the antibiotic eluted in situ from bone cement which is found to exceed 100–1000 fold the minimum inhibitory concentration (MIC), the microbial load is intended to be reduced [55]. Although our study reveals high resistance rates among MRSA (76%), MRSE (75%) and SE (52%) pathogens to clindamycin, the synergistic interplay with gentamicin and the initial high dose released from ALBCS may still facilitate adequate pathogen eradication in these instances. Systemic clindamycin administration in PJI should be critically evaluated in the presence of high resistance rates although there was a moderate linear downward trend (r^2 ^= 0.3868) over time in our study.

Vancomycin is a widely used antibiotic for the treatment of serious Gram-positive bacterial infections. It is also among the first line treatment for infections caused by MRSA [56]. According to clindamycin vancomycin is frequently administered systemically and in ALBCS in PJI with high success rates [57]. However, vancomycin therapy shows a narrow therapeutic range due to serious adverse reactions such as acute kidney injury (AKI) and ototoxicity [58]. The number of PJIs caused by resistant germs, including vancomycin-resistant *staphylococcus aureus* (VRSA) and VRE is increasing [50]. In our study, the overall resistance rate against vancomycin was less than 1% with almost no linear trend over time. (r^2 ^= 0.0692). As expected, *enterococci* showed the highest resistance rate with around 4% followed by MRSE with nearly 1%. The frequently reported low resistance rates make vancomycin very suitable to act as antibiotic systemic agent and in ALBCS. It remains to be seen whether MRSE germs continue to increase and whether their resistance level against vancomycin changes.

Gentamicin plays a crucial role in PJI in ALBCS against GN bacteria and *staphylococci* while it is almost never used systemically. Corona et al. found no superiority of vancomycin-gentamicin combination spacers over gentamicin alone [59]. Our study reveals gentamicin resistance in 29.4% of pathogens with weak linear downward trend over time (r^2^=0.2115). This raises questions regarding the efficacy of gentamicin monotherapy in spacers despite the high locally applied dosage of the ALBCS. Notably, MSSA and MRSA exhibit high sensitivity rates (97.4 and 89.5%, respectively). However, gentamicin resistance is alarmingly high in *staphylococcus epidermidis* (43%) and particularly in MRSE (64.6%). These findings suggest potential adjustments to spacer therapy components, such as integrating additional antibiotics or modifying dosages, to ensure effective pathogen eradication. The dose-dependent nephro- and ototoxicity of gentamicin necessitates again alertness, particularly in elderly or renally impaired patients [60].

Fluoroquinolones (FQ) such as ciprofloxacin, are the first choice as anti-biofilm antibiotics for the treatment of GN-PJI [61]. However, FQ show severe complications including, tendinopathy, peripheral neuropathy, nausea, or diarrhea that were observed in almost 20% of patients [62]. In addition, the increasing of resistance to fluoroquinolones and aminoglycosides leads to impaired treatment options for GN infections [63]. In our study, the overall resistance rate against FQ was high with 37.3% with a moderate decline over time (r^2 ^= 0.4765). In a meta-analysis, the prevalence of resistance in selected gram-positive bacterial isolates against ciprofloxacin was described to be nearly 20% [64]. Astonishingly, in our study the resistance rate against FQ of GN pathogens (19.3%) was higher than in MSSA (11.8%) and MSSE (15.0%). The highest resistance rates were found on MRSA (81.3%), SE (48.2%) and MRSE (84.0%).

Rifampicin has attracted attention for its unique pharmacokinetic properties and ability to penetrate biofilms, making it a potentially valuable option in the treatment of PJIs, but must be used in combination due to the high prevalence of resistance development [65]. Evidence to support rifampicin combination therapy is conflicting and limited [66]. Rifampicin’s adverse effects and drug interaction profiles further complicate its use as a long-term biofilm eradication treatment [67]. In our study, the overall resistance rate was low with 5.7% with no linear trend over time (r^2 ^= 0.0229). The highest resistance rates were shown in SE (7.1%), MRSA (11.8%) and MRSE (13.6%).

Cefazolin is a first-generation cephalosporin broad-spectrum antibiotic for the treatment of serious gram-positive and gram-negative infections [67]. It is used as a prophylaxis antibiotic before performing a wide range of surgical operations [68]. Cefazolin is associated with rare and mild adverse effects such as urticaria, skin reaction, diarrhea, vomiting, and transient neutropenia, rarely leading to life threatining situations [69]. Cefazolin can only be administered intravenously making it necessary to switch to another oral antibiotic or to perform an outpatient intravenous application using peripherally inserted central catheters (PICCS)[70].. Beyond, cefazolin only penetrates poorly through blood-bone barrier (~ 20%) [71].

Linezolid is a potential antimicrobial treatment option addressing resistant *staphylococci* [72]. It reduces the need for long-term inpatient treatment given its excellent oral bioavailability and moderate bone penetration (serum/bone ratio 40–51%) [71]. However, linezolid can have some feared adverse effects such as cytopenia, particularly of leukocytes and neuropathy, that might lead to treatment discontinuation. Longer courses of treatment require close surveillance, and patients at risk of non-optimal dosage should undergo drug monitoring [73].

Meropenem, a broad spectrum carbapenem antibiotic, has excellent penetration capabilities, allowing it to reach various tissues and biological compartments, including the joint cavity. This characteristic is essential for the drug to effectively target and eliminate bacterial pathogens at the site of infection. However, the stability of meropenem is lower than other antibiotics, resulting in faster degradation and a shorter half-life [74, 75] and the bone penetration is low (serum/bone ration ~20%) [71]. It was shown that vancomycin combined with carbapenems achieved effective coverage rates in chronic PJI patients [76].

*Pseudomonas aeruginosa* (PA) is a particularly challenging pathogen to treat due to emerging multi-drug resistant strains [77]. Piperacillin, often administered in combination with tazobactam, is a key drug in the treatment of PA infections [78]. Piperacillin has the best effect when free drug concentration is above the minimal inhibitory concentration at target sites [79]. Piperacillin continuous infusion resulted in steady state concentration compared to intermittent short-term infusion while treating PA PJI [80].

Daptomycin (DAP) is a novel option for PJI treatment because it exerts bactericidal activity against gram-positive bacteria, including multiple-resistant isolates and stationary-phase bacteria in biofilm present on implants [81]. DAP shows high penetration into biofilms, low bactericidal concentration, and synergistic effects with rifampicin [82]. However, DAP sparsely penetrates into bone (~ 7%) [71, 83, 84].

The study has some limitations. Due to the retrospective design and the long study period new features and resistant profiles in microbiological diagnostics could not be applied to all analyses. Especially, the EUCAST criteria have been revised in recent years with adjusted doses for antimicrobial substances and limit values. Consequently, the results are not all comparable with one another. The heterogeneity of cases with acute and chronic PJI was not precisely determined. Further, we did not correlate our microbiological results with clinical outcome.

## Conclusion

The current study reveals disturbingly high rates of MRSE, SE and GN pathogens causing PJI. GN and MRSE rates were also increased in PM PJI. The shoulder joint is a predilection site for PJI caused by *Cutibacteria*. The high overall resistance rates against ampicillin/sulbactam raise doubts as first line empiric oral antibiotic in PJI. The nearly 20% ciprofloxacin resistant gram-negative pathogens should be alarming due to limited choice of biofilm-penetrating antibiotics in gram-negative PJI.

## Data Availability

The data were gathered from the archives of the participating hospitals.

## Abbreviations

PJI: Periprosthetic joint infection
MSSA: Methicillin-sensitive *Staphylococcus aureus*
MRSA: Methicillin-resistant *Staphylococcus aureus*
MSSE: Methicillin-sensitive *Staphylococcus epidermidis*
MRSE: Methicillin-resistant *Staphylococcus epidermidis*
PM: Polymicrobial
GPR: Gram-positive rods
GN: Gram-negative
SE: Streptococcus and enterococcus
CN: Culture negative
VRE: Vancomycin-resistant enterococci
ESBL: Extended-spectrum beta-lactamases
C. acnes: Cutibacterium acnes
TJA: Total joint arthroplasty
DAA: Direct anterior approach
ALBCS: Antibiotic loaded bone cement spacers
FQ: Fluoroquinolones
